# Leading with AI in critical care nursing: challenges, opportunities, and the human factor

**DOI:** 10.1186/s12912-024-02363-4

**Published:** 2024-10-14

**Authors:** Eman Arafa Hassan, Ayman Mohamed El-Ashry

**Affiliations:** 1 Critical Care and Emergency Nursing Department, Faculty of Nursing, Alexandria University, Alexandria, Egypt; 2 Psychiatric and Mental Health Nursing Department, Faculty of Nursing, Alexandria University, Alexandria, Egypt; 3 Psychiatric and Mental Health Nursing, Department of Nursing, College of Applied Medical Sciences, Jouf University, Al-Qurayyat, Saudi Arabia

**Keywords:** Artificial intelligence, Critical care nursing, Ethical issues, Human factors, Nurse-computer interface

## Abstract

**Introduction:**

The integration of artificial intelligence (AI) in intensive care units (ICUs) presents both opportunities and challenges for critical care nurses. This study delves into the human factor, exploring how nurses with leadership roles perceive the impact of AI on their professional practice.

**Objective:**

To investigate how nurses perceive the impact of AI on their professional identity, ethical considerations surrounding its use, and the shared meanings they attribute to trust, collaboration, and communication when working with AI systems.

**Methods:**

An interpretive phenomenological analysis was used to capture the lived experiences of critical care nurses leading with AI. Ten nurses with leadership roles in various ICU specializations were interviewed through purposive sampling. Semi-structured interviews explored nurses’ experiences with AI, challenges, and opportunities. Thematic analysis identified recurring themes related to the human factor in leading with AI.

**Findings:**

Thematic analysis revealed two key themes which are leading with AI: making sense of challenges and opportunities and the human factor in leading with AI. The two main themes have six subthemes which revealed that AI offered benefits like task automation, but concerns existed about overreliance and the need for ongoing training. New challenges emerged, including adapting to new workflows and managing potential bias. Clear communication and collaboration were crucial for successful AI integration. Building trust in AI hinged on transparency, and collaboration allowed nurses to focus on human-centered care while AI supported data analysis. Ethical considerations included maintaining patient autonomy and ensuring accountability in AI-driven decisions.

**Conclusion:**

While AI presents opportunities for automation and data analysis, successful integration hinges on addressing concerns about overreliance, workflow adaptation, and potential bias. Building trust and fostering collaboration are fundamentals for AI integration. Transparency in AI systems allows nurses to confidently delegate tasks, while collaboration empowers them to focus on human-centered care with AI support. Ultimately, dealing with the ethical concerns of AI in ICU care requires prioritizing patient autonomy and ensuring accountability in AI-driven decisions.

**Supplementary Information:**

The online version contains supplementary material available at 10.1186/s12912-024-02363-4.

## Introduction

Critical care units are complex environments demanding high-acuity decision-making and efficient resource allocation. Artificial Intelligence (AI) has the potential to revolutionize critical care by automating tasks, providing real-time data analysis, and supporting clinical decision-making [1]. The rise of AI in healthcare presents both exciting opportunities and significant challenges. AI algorithms can analyze vast amounts of patient data, identify trends, and offer real-time decision support tools. This has the potential to improve patient outcomes, streamline workflows, and free up time for nurse leaders to focus on more complex tasks.

However, concerns exist regarding the limitations of AI, particularly its reliance on data quality and the potential for bias in algorithms. Nurse leaders also face challenges integrating AI into existing workflows and ensuring they have the skills and knowledge to effectively utilize these new tools [2, 3]. Critical care nurse leaders have an important role in leveraging AI technologies to enhance patient outcomes [4]. These leaders are instrumental in overseeing the implementation of AI-driven tools that assist in monitoring patient vitals, predicting potential complications, and optimizing resource allocation. For instance, AI algorithms can analyze vast amounts of data to identify early signs of sepsis or other critical conditions, allowing for more proactive and effective interventions [5].

Despite the potential benefits, the attitudes of critical care nurse leaders toward AI are mixed, influenced by factors such as perceived reliability, ethical considerations, and the potential impact on the nurse-patient relationship. Some nurse leaders express optimism about the ability of AI to enhance clinical decision-making and improve patient safety [6]. However, there are concerns regarding the integration of AI into existing workflows, the need for adequate training, and the potential for job displacement. Moreover, ethical issues surrounding data privacy and the transparency of AI decision-making processes remain significant barriers to widespread acceptance. Consequently, while there is cautious optimism about the role of AI in critical care, addressing these concerns through robust training programs and clear ethical guidelines is essential for its successful integration [7].

Understanding nurse leaders’ perspectives on AI is important for promoting successful adoption and optimizing the human-AI partnership in critical care settings. A growing body of research explores the potential of AI in healthcare, with studies showing that AI can be effective for tasks such as analyzing patient data, predicting patient outcomes, and providing early warnings of potential complications. However, research also highlights the challenges of AI adoption, including concerns about data privacy, security, and potential biases in algorithms [8–10]. Leadership in the context of AI integration is an emerging area of inquiry. Limited research explores the specific challenges and opportunities faced by nurse leaders as they adapt to working with AI tools [4, 11]. This study aims to contribute to this area by exploring the perspectives of critical care nurse leaders on their evolving role in an AI-driven environment.

## Methods

### Design

This research design employs Interpretive Phenomenological Analysis (IPA) to capture the lived experiences of critical care nurses leading with AI [12]. IPA aligns perfectly with this study’s aim as it emphasizes understanding the subjective meaning nurses make of this new technology in their work environment [13]. By taking an idiographic approach, IPA allows for exploration of the unique experiences of individual nurses. This delves deeper than simply reporting factual events, uncovering the richness and complexity of their perspectives on challenges, opportunities, and the human factor in AI-assisted critical care. IPA’s focus on lived experience makes it particularly suited to capture the human element in this context [12]. In-depth interviews and thematic analysis, hallmarks of IPA, helped us unveil the deeper meanings attached to these experiences, encompassing not just factual accounts but also other aspects of leading with AI.

### Participants and context

This study employed a purposive sampling strategy to recruit critical care nurses with in-depth experience and leadership roles. This ensured participants could provide rich insights into the impact of AI on their professional identity, ethical considerations, and collaboration dynamics [14]. We recruited ten nurses with a minimum of two years of experience in critical care settings, specifically targeting those holding leadership positions within critical care units such as nurse managers, head nurses, and in-charge nurses as illustrated in Table 1. This combination of experience and leadership allowed participants to offer a broader perspective on unit workflow and how AI integration potentially affects team dynamics and patient care delivery. All participants were required to have experience or exposure to AI tools and technologies used in healthcare settings. This ensured they could directly address the research questions related to trust, collaboration, and communication with AI systems.

**Table 1:** Nurses’ characteristics

Nurse ID	Age	Gender	ICU specialization	Leadership role
1	45	Female	General Adult ICU	Nurse Manager
2	38	Male	Surgical ICU	Head Nurse
3	42	Female	Medical ICU	In-charge Nurse
4	48	Female	Coronary Care Unit	Nurse Manager
5	35	Male	Trauma ICU	Head Nurse
6	52	Female	General Adult ICU	Head Nurse
7	30	Male	General Adult ICU	Nurse Manager
8	32	Female	Medical ICU	Head Nurse
9	37	Male	Coronary Care Unit	In-charge Nurse
10	50	Female	Trauma ICU	Head Nurse

To reach a diverse sample of participants, we utilized a multi-pronged approach. Online recruitment allowed targeted outreach based on participant criteria. Informational letters with study details were disseminated through WhatsApp lists of relevant healthcare institutions. Additionally, snowball sampling was employed, where nurses working in participating ICUs identified colleagues who met the study criteria. This approach tapped into existing professional networks and reached a wider pool of potential participants [15].

The study included critical care nurses from various intensive care unit (ICU) specializations at four different hospitals. These specializations included General Adult ICU, Coronary Care Unit, Surgical ICU, Medical ICU, and Trauma ICU. This diversity in ICU types allowed for a broader understanding of how AI integration impacts critical care practices across different patient populations and care settings.

### Data collection

This study employed in-depth, semi-structured, one-on-one interviews to collect rich and detailed data from critical care nurses. This approach aligns with the IPA design, allowing participants to share their lived experiences and perspectives on leading with AI in critical care settings [12, 16]. Following written informed consent, participants engaged in individual virtual interviews lasting 60–90 min. All ethical guidelines were strictly adhered to, ensuring participants’ confidentiality and the secure handling of data [17].

A pre-developed interview guide served as a framework to explore the research questions through open-ended prompts as a supplementary file 1. The guide was developed based on a review of relevant literature and expert consultations to ensure it effectively addressed the study’s aims [18]. Examples of prompts included: “Can you describe how AI tools have impacted your role as a critical care nurse leader?” or “Have you encountered any ethical dilemmas related to using AI in patient care?”

The interviewer actively thought and encouraged participants to elaborate on their experiences, delve deeper into their thoughts, and raise any additional points they felt were important. This flexible approach allowed the interview to follow the participant’s lead while ensuring all key areas were addressed [19]. The interviews were audio-recorded verbatim with participant consent to capture all nuances of the conversation. The recordings were then transcribed for analysis, ensuring accuracy and completeness [20]. Ten interviews continued until data saturation was achieved, meaning no new themes or insights were emerging from subsequent interviews [21].

### Data analysis

This study employed a six-step, iterative, and inductive approach to analyze interview data from critical care nurses, following the principles of Interpretative Phenomenological Analysis (IPA) outlined by Smith et al., (2009) [12]. In the first step, researchers began by deeply immersing themselves in the data through meticulous reading and rereading of interview transcripts. This fostered a familiarity with the content and nuances of participants’ narratives related to their experiences with AI integration in critical care settings.

Transcripts were then subjected to initial noting, where researchers made descriptive, linguistic, and conceptual comments. Descriptive comments (normal text) captured the core content of nurses’ statements regarding AI use, challenges, and opportunities. Linguistic comments (italics) focused on exploring the specific language used by participants when discussing AI, such as metaphors or technical jargon. Conceptual comments (underlined) aimed to delve deeper, engaging with participants’ meaning-making processes around AI’s impact on their professional identity, collaboration dynamics, and patient care within their specific ICU environments.

Analyzing the initial notations shifted the focus to identifying recurring themes within each transcript. This process involved close attention to both individual experiences with AI and the broader context of participants’ ICU roles and leadership responsibilities. The identified themes went beyond a chronological listing; they captured the essence of participants’ experiences leading with AI in critical care settings.

Once themes were established within each interview, the next step involved exploring how they connected and fit together across cases. This involved a process of abstraction, where similar themes related to AI’s impact were grouped and given new labels reflecting their combined essence. For instance, themes around “distrust in AI decision-making” and “concerns about patient safety” were grouped under a broader theme of “ethical considerations in AI use.” Subsumption, a related process, explored how certain themes might encompass or subsume related themes. For example, a theme of “increased workload due to AI” is subsume themes of “frustration with troubleshooting AI errors” and “challenges integrating AI into existing workflows.”

While a single case study could be written after analyzing one interview, IPA typically involves multiple participants. The process of initial noting, theme identification, and exploration of connections was then repeated for each subsequent interview transcript. Importantly, each case was treated on its own merits, allowing new themes to emerge related to leading with AI, while maintaining the rigor of the established analytical framework.

The final step involved a cross-case analysis, searching for overarching patterns and connections across all interviews. Researchers compared the thematic maps from each case to identify how themes resonated or differed between participants in various ICU specializations. This comparison might lead to further refinement and relabeling of themes to capture the full breadth of experiences with leading AI in critical care across the study as a supplementary file 2.

### Trustworthiness and rigor

To further enhance trustworthiness, we conducted member checking by sharing our initial thematic interpretations with two nurses to ensure they resonated with their experiences. Their feedback confirmed the accuracy of our interpretations and provided valuable insights that enriched our understanding. Additionally, we acknowledged the potential for researcher bias and maintained a detailed audit trail to document all research decisions, including interview protocols, coding schemes, and any revisions made throughout the analysis process. For rigor, intercoder reliability was established as the two authors were involved in data analysis. This involved comparing coding between authors to ensure consistency and minimize subjective bias in the analysis. Furthermore, an expert in qualitative research reviewed our cross-case analysis to ensure the validity and reliability of our findings.

### Ethical considerations

Ethical approval was obtained from the Research Ethics Committee at the Faculty of Nursing, Alexandria University (IRB00013620). All participants voluntarily participated in the study after a detailed explanation of the study’s purpose. Their right to withdraw from the study at any time was emphasized. To ensure confidentiality, interviews were anonymized during transcription and data analysis. With participant consent, interviews were audio-recorded to capture the entire conversation for accurate analysis. Secure storage protocols were employed for all audio recordings and transcripts to minimize the risk of unauthorized access.

## Finding

Findings from the study investigate how critical care nurses make sense of the challenges and opportunities associated with leading with AI in their practice. Thematic analysis was conducted on interview data collected from ten critical care nurses. The analysis identified two main themes which are leading with AI: making sense of challenges and opportunities (theme 1) and the human factor in leading with AI (theme 2).

### Leading with AI: making sense of challenges and opportunities

This theme explored how critical care nurses are making sense of the challenges and opportunities associated with leading with AI in their practice. Interviews revealed that nurses are interpreting the impact of AI on their daily work in various ways. Some described a positive influence on decision-making through access to real-time data analysis and early warning scores. Conversely, others expressed concerns about overreliance on AI and the need to maintain their clinical expertise. Furthermore, the theme highlighted a potential shift in roles and responsibilities, with some nurses mentioning a supervisory role focused on overseeing AI outputs and others encountering new responsibilities like managing AI systems. While workload changes varied, all participants emphasized the importance of ongoing training and support to effectively utilize AI in critical care.

#### Meaning-making of impact on practice

Critical care nurses in this study described the impact of AI on their practice in various ways. Some nurses highlighted the benefits of AI for automating routine tasks, such as generating early warning scores or compiling patient data summaries. This freed up time for nurses to focus on more complex aspects of patient care, such as emotional support and individualized treatment plans.

“*AI…frees me up to spend more time with patients*,*….It lets me focus…like talking to patients and creating care plans.*” Nurse number 7.

However, some nurses also expressed concerns about overreliance on AI and the potential for AI-generated alerts to lead to information overload. They emphasized the importance of maintaining their clinical judgment and critical thinking skills when using AI to make patient care decisions.

“……. *AI helps with routine tasks*,* but the constant alerts can be overwhelming. The key is using it effectively*,* not replacing my critical thinking.*” Nurse number 2.

#### Shifting roles and responsibilities

Interviews with critical care nurses revealed a spectrum of perspectives regarding the evolving roles and responsibilities associated with leading with AI. Some nurses described a shift towards a more supervisory role, focusing on overseeing AI outputs and ensuring their validity before integrating them into patient care decisions.

“*With AI*,* my role is more oversight*,* ……. I ensure AI outputs make sense before using them in patient care.*” Nurse number 8.

Nevertheless, many nurses also acknowledged the potential for AI to augment their expertise by providing real-time data analysis and facilitating earlier identification of patient deterioration. They emphasized the importance of adapting their skillsets to leverage the strengths of AI while maintaining their core competencies in critical thinking, communication, and patient advocacy.

“*AI enhances our expertise. It analyzes data*,* flags issues early*,* and frees me to focus on patient care. We need to adapt*,* not replace*,* our skills. Critical thinking*,* communication*,* and advocacy remain essential.*” Nurse number 6.

#### Workload and workflow changes

Nurses expressed concerns about the increased workload associated with learning and integrating new AI tools into their workflow. Troubleshooting technical issues and adapting to new routines could create initial challenges. Additionally, some nurses mentioned the potential for information overload due to the constant stream of data generated by AI systems.

“*Troubleshooting and data overload are initial challenges. We need to balance learning with managing data effectively.*” Nurse number 1.

Interviews also revealed the importance of clear communication and collaboration among nurses, other healthcare professionals, and IT staff for a smooth transition to AI-integrated workflows. Nurses who felt adequately supported during AI implementation reported a more positive experience with workload adjustments.

“*Clear communication and teamwork are important for supporting AI integration. ……. The support from other healthcare and IT personnel during implementation helps manage workload adjustment.*.” Nurse number 5.

### The human factor in leading with AI

This theme explored the critical role human factors play in leading with AI. Nurses described a range of experiences regarding trust in AI, emphasizing transparency as key for building trust in AI outputs. Collaboration emerged as an important factor, with nurses highlighting the value of AI for tasks like data analysis, allowing them to focus on human-centered aspects of care. Ethical considerations were also prominent, with concerns about potential bias in AI algorithms and the need for clear accountability in AI-driven decisions. Furthermore, maintaining patient autonomy remained a priority, emphasizing the irreplaceable role of nurses in patient advocacy. Communication with AI systems presented both challenges and successes. While some nurses encountered difficulties due to unclear explanations or limited communication channels, others reported positive experiences with clear and transparent AI outputs, fostering trust and smoother workflow integration.

#### Building trust and collaboration

Critical care nurses in this study expressed a range of experiences regarding building trust and collaborating with AI tools. Some nurses emphasized the importance of understanding how AI systems work and the data they use to generate outputs. This transparency fostered a sense of trust and allowed nurses to interpret AI recommendations critically and integrate them effectively into their decision-making processes.

“*Trusting AI requires understanding how it works…….Transparency in data and analysis allows critical evaluation and integration of AI recommendations into patient care.*” Nurse number 9.

However, others expressed concerns about the black-box nature of some AI algorithms, making it difficult to understand the rationale behind certain suggestions. This lack of transparency could hinder trust and limit nurses’ willingness to rely on AI outputs.

“*AI suggestions feel like magic tricks. Lack of transparency hinders trust. We need to see the data and reasoning behind recommendations to collaborate effectively*.” Nurse number 5.

Collaboration with AI emerged as an important human factor for many nurses. They described successful collaboration when AI handled tasks like data analysis and generating early warning scores, allowing nurses to focus on patient interaction and applying their clinical expertise. Effective communication between nurses and AI systems was also highlighted as central for seamless collaboration.

“*……………. AI handles data*,* nurses focus on patients. Clear communication of these data is key for collaboration to provide the best care.*” Nurse number 8.

#### Ethical considerations and meaning-making

Critical care nurses in this study identified various ethical dilemmas associated with AI use in critical care. Some nurses expressed concerns about potential bias within AI algorithms, particularly if the training data used to develop the AI lacked diversity or reflected historical biases in healthcare. This could lead to unfair treatment decisions for certain patient populations.

*“AI bias worries me. Biased training data can lead to unfair care. We need diverse data to ensure AI fairness.*” Nurse number 9.

Interviews also revealed the importance of maintaining patient autonomy in the age of AI. Nurses emphasized the need to ensure that AI did not replace their role in advocating for patients and ensuring their wishes were considered in treatment decisions.

“*Patient autonomy is key………AI informs*,* but doesn’t replace*,* advocating for patients’ wishes in care.*” Nurse number 7.

#### Communication and transparency

Critical care nurses in this study described a mixed bag of experiences regarding communication and transparency with AI systems. Some nurses highlighted the challenges of understanding complex AI outputs. The lack of clear explanations for AI-generated recommendations could lead to confusion and hinder nurses’ ability to effectively utilize the information in patient care decisions.

“*AI data and reports are sometimes confusing. We need clear explanations in plain language to use AI effectively in patient care.*” Nurse number 10.

However, other nurses reported positive experiences when AI systems provided clear and concise explanations for their outputs. This transparency enhanced trust and allowed nurses to integrate AI recommendations more seamlessly into their workflow. Effective communication design within AI interfaces was also identified as a factor contributing to successful communication with these systems.

“*Clear explanations and user-friendly interfaces build trust and make integrating AI recommendations easier*.” Nurse number 8.

## Discussion

Introducing AI into critical care has significantly impacted how nurses perform their duties. The study highlights both positive and negative aspects of this technological advancement. On the positive side, AI enhances decision-making capabilities by providing real-time data analysis and early warning scores. This aligns with findings by Gallo et al., (2024) [22], which underscore AI’s potential to improve patient outcomes through early intervention and reduce the burden of routine tasks on healthcare professionals. Arnold et al., (2019) [23] highlighted that AI could process vast amounts of data more efficiently than humans, supporting timely and accurate clinical decisions.

However, the current study revealed concerns about overreliance on AI and the potential erosion of clinical skills. This sentiment is reflected in the literature, where apprehensions about “de-skilling” healthcare professionals have been documented [24]. Nurses in our study stressed the importance of maintaining clinical expertise alongside AI use, highlighting the need for balanced integration. This is consistent with Aquino et al., (2023) [25] views, who argue for a synergistic approach where AI complements but does not replace human judgment. A study conducted by Amann et al., (2020) [26] also supports the need for balanced integration, emphasizing the importance of human oversight in AI-driven healthcare to mitigate risks and enhance patient safety.

The shifting roles and responsibilities identified in the study reflect a broader trend in healthcare where technology necessitates new skill sets and professional roles [27]. The study participants described a transition towards supervisory roles overseeing AI outputs and new responsibilities such as managing AI systems. This transition is supported by findings from Bajwa et al. (2021) [28], who noted that integrating AI in healthcare requires continuous professional development and adaptation to new workflows. Moreover, AI’s impact on workflow is confirmed by Fogel and Kvedar (2018) [29], who discussed how AI could redistribute workloads, allowing healthcare professionals to focus more on patient-centric tasks.

The second theme underscores the critical role of human factors in successfully integrating AI into nursing practice. Trust in AI emerged as a significant factor, with transparency being the key to building this trust. The literature supports this finding, emphasizing the importance of explainable AI in healthcare to ensure transparency and trustworthiness [30]. Nurses expressed concerns about the “black box” nature of some AI systems, which can obscure the decision-making process and hinder trust. Lipton (2018) [31] and Gunning et al. (2019) [32] highlight that for AI to be effectively integrated into clinical practice, it must provide clear and interpretable explanations of its outputs. The need for transparency is further supported by a study conducted by Binns et al. (2018) [33], which found that healthcare professionals are likelier to trust and utilize AI systems that provide understandable and transparent decision-making processes.

Collaboration between nurses and AI systems was another critical aspect that emerged in the study. Effective collaboration allows nurses to leverage AI for tasks such as data analysis, freeing them to focus on patient-centered care. This collaborative approach is advocated by Topol (2019) [3], who suggests that AI can enhance human capabilities in healthcare by handling data-intensive tasks. For this collaboration to be effective, clear communication channels between AI systems and healthcare professionals are essential, as Reddy, Fox, and Purohit (2019) [34] noted. Effective collaboration is further emphasized by Liu et al. (2020) [35], who demonstrated that successful AI integration in healthcare settings relies on well-designed interfaces and communication strategies that align with clinical workflows.

The current study participants raised ethical concerns regarding AI in critical care, particularly around potential biases in AI algorithms and accountability in AI-driven decisions. These concerns are well-documented in the literature. Obermeyer et al. (2019) [36] and Nazerid et al., (2023) [8] discuss how biases in training data can lead to biased AI systems, which can perpetuate healthcare disparities. Ensuring that AI systems are trained on diverse and representative data is crucial to mitigate these biases and promote fairness in AI-driven healthcare decisions. A study by Ueda et al., (2023) [37] further reinforced the importance of addressing bias, which found that biased AI algorithms could lead to significant disparities in healthcare outcomes.

Accountability is another ethical issue highlighted by the study participants. The question of who is responsible for decisions influenced by AI is complex and multifaceted. This concern is reflected in the work of Elendu et al., (2023) [38], who argue that clear guidelines and accountability frameworks are necessary to delineate responsibilities in AI-assisted medical decision-making. Transparent decision-making processes are essential to ensure accountability and trust in AI systems. A study by Morley et al. (2020) [7] supports this by highlighting the need for ethical frameworks that clearly define accountability in AI-driven healthcare.

Interviews also revealed the importance of maintaining patient autonomy in the age of AI. Nurses emphasized the need to ensure that AI did not replace their role in advocating for patients and ensuring their wishes were considered in treatment decisions. This aligns with patient-centered care principles, prioritizing patient autonomy and individualized treatment plans [39]. The importance of maintaining patient autonomy is further underscored by a review conducted by Pierre et al., (2023) [40], which found that patient-centered AI systems can be more effective in improving patient satisfaction and outcomes.

Effective communication and ongoing training are critical for integrating AI into nursing practice. The study highlights the importance of clear explanations for AI-generated recommendations and robust communication channels. These findings are supported by Grote and Berens (2020) [41], who emphasize that training healthcare professionals to understand and work with AI is vital for successful integration. Continuous professional development programs that focus on AI’s technical and ethical aspects can help nurses adapt to new technologies and leverage them effectively in their practice. A study by Holzinger et al. (2020) [42] suggests that interdisciplinary training programs that include both technical and clinical education can significantly enhance the integration of AI in healthcare.

The mixed experiences of nurses regarding communication and transparency with AI systems highlight the need for user-friendly AI interfaces that provide clear and concise information. As Sendak et al. (2020) [43] noted, effective AI tools in healthcare should be designed with end-users in mind, ensuring that outputs are easily interpretable and actionable. The positive experiences reported by some nurses when AI systems provided clear explanations underscore the importance of designing AI tools that prioritize usability and transparency.

Despite adhering to the rigorous qualitative research methodology of interpretive phenomenology and conducting the study across four hospitals to capture diverse nursing experiences, this research still has several limitations. The sample size of ten participants may restrict the generalizability of the findings to a broader population of critical care nurses. To enhance the applicability of the results, future studies could benefit from a larger sample size. Additionally, further research exploring the perspectives of bedside nurses who interact directly with AI could reveal challenges and opportunities that might not be evident from a leadership standpoint.

## Conclusion

While nurses acknowledged AI’s potential to streamline workflows, improve data analysis, and facilitate early patient deterioration detection, concerns arose regarding workload, information overload, and difficulties interpreting AI outputs. The key to unlocking AI’s potential lies in addressing these human factors. Clear and concise communication from AI systems, with user-friendly interfaces and transparent explanations for recommendations, is crucial for building trust and fostering collaboration. Additionally, ensuring fairness and mitigating potential bias within AI algorithms is essential for ethical patient care.

## Electronic supplementary material

Below is the link to the electronic supplementary material.

Supplementary Material 1

Supplementary Material 2

## Data Availability

The data and materials of the current study are not publicly available due to confidentiality reasons but are available from the corresponding author on reasonable request.
