# Supplementary material for: Leading with AI in critical care nursing: challenges, opportunities, and the human factor
Source: BMC Nurs. 2024 Oct 14;23:752. doi: 10.1186/s12912-024-02363-4 (PMC11475860; doi:10.1186/s12912-024-02363-4)
Supplement: Supplementary file 1 — Supplementary Material 1 [file 12912_2024_2363_MOESM1_ESM.docx]

**Example of thematic map during data analysis**

**Examples of Sub-themes from Participant Number 3**

- **Challenges**
  - Increased workload due to AI
  - Technical difficulties in AI integration
  - Resistance from colleagues
  - Concerns about job security
- **Opportunities**
  - Improved efficiency
  - Enhanced decision-making
  - Positive impact on patient outcomes

**Cross-Case Analysis and Comparison**

By comparing thematic maps, researchers identified the following patterns:

- **Theme Consistency**

Example: The subtheme of "Increased workload due to AI" appears in multiple cases, suggesting it's a common challenge across different ICU specializations.

- **Theme Variation**

Example: The subtheme of "Positive impact on patient outcomes" is more prevalent in the Coronary Care Unit case, indicating that the benefits of AI may vary depending on the specific ICU setting.

- **Theme Refinement**

Example: The subtheme of "Challenges in integrating AI into existing workflows" was refined to include other sub-themes like "Technical difficulties" and "Resistance from colleagues" based on the specific challenges mentioned in different cases.

**Example of a Version of Refined Themes and Sub-themes**

- **Making Sense of Challenges and Opportunities**
  - Meaning-Making of Impact on Practice
    - Increased Efficiency and Reduced Workload
    - Concerns about Overreliance on AI
  - Shifting Roles and Responsibilities
    - Supervisory Role
    - Augmented Expertise
  - Workload and Workflow Changes
    - Initial Challenges
    - Importance of Support and Communication
- **The Human Factor**
  - Building Trust and Collaboration
    - Understanding AI Systems
    - Transparency and Communication
  - Ethical Considerations
    - AI Bias
    - Patient Autonomy
  - Communication with AI Systems
    - Challenges in Understanding
    - Importance of Clear Explanations

**Connecting Lines**

- Meaning-Making of Impact on Practice to Shifting Roles and Responsibilities

Nurses' experiences with AI's impact on their workload and efficiency influenced their changing roles and responsibilities.

- Building Trust and Collaboration to Ethical Considerations

Trust in AI is essential for nurses to navigate ethical dilemmas and ensure patient autonomy.

- Communication with AI Systems to Meaning-Making of Impact on Practice

Clear communication with AI systems can enhance nurses' understanding of AI outputs and their ability to integrate them into their practice.
