# Supplementary material for: Leading with AI in critical care nursing: challenges, opportunities, and the human factor
Source: BMC Nurs. 2024 Oct 14;23:752. doi: 10.1186/s12912-024-02363-4 (PMC11475860; doi:10.1186/s12912-024-02363-4)
Supplement: Supplementary file 2 — Supplementary Material 2 [file 12912_2024_2363_MOESM2_ESM.docx]

**Interview Guide: Leading with AI in Critical Care Nursing: Challenges, Opportunities, and the Human Factor**

**Demographic and Background Information**

- Nurse Participant ID ……………………..
- Age …………………..
- Gender ……………………….
- Highest Level of Education (BSN, MSN, PhD)
- Years of Experience as a Nurse ……………………….
- Years of Experience in Critical Care Nursing ………………………
- Current Leadership Role in Critical Care Unit (Nurse Manager, head nurse, in charge nurse)
- Experience with specific AI tools
- Clinical decision support systems (CDSS)
- AI-powered diagnostic tools
- Chatbots
- Virtual assistants for patient monitoring or data entry

**Interview Questions**

- Warm-up: Can you tell me a bit about your experience as a critical care nurse leader?
- Experience with AI: Have you encountered any AI tools or systems within your critical care unit? (If yes) "Can you briefly describe the specific type of AI you've interacted with?

**Professional Identity & Sense of Purpose:**

- **Can you describe how AI tools have impacted your role as a critical care nurse leader?** (Probe for positive or negative impacts)
- In your experience, has AI made patient care more efficient within your unit? How has this affected the overall sense of purpose among your team?
- Are there any aspects of critical care leadership that you believe cannot be replaced by AI? (Why or why not?)

**Ethical Considerations:**

- **Have you encountered any ethical dilemmas related to using AI in patient care?** (Provide examples if needed: "e.g., bias in decision-making, transparency of AI outputs)
- How do you feel about the potential for AI bias impacting patient care within your unit? (Examples?)
- Who should be ultimately responsible for patient care decisions in an AI-integrated environment - the nurse leader or the AI system? (Why?)

**Trust, Collaboration & Communication:**

- In an ideal scenario, how would critical care nurses and AI systems collaborate to optimize patient care within your unit?
- What factors would contribute to your trust in an AI system for critical care tasks? (Transparency, Explainability?)
- How do you envision effective communication happening between nurses and AI in the ICU? (Examples?)
